# Monte Carlo method for assessment of a multimodal insertable biosensor

**DOI:** 10.1117/1.JBO.27.8.083017

**Published:** 2022-05-03

**Authors:** Jesse Fine, Michael J. McShane, Gerard L. Coté

**Affiliations:** a Texas A&M University, Department of Biomedical Engineering, College Station, Texas, United States; b Texas A&M University, Department of Materials Science and Engineering, College Station, Texas, United States; c Texas A&M University, Center for Remote Health Technologies and Systems, Texas A&M Engineering Experiment Station, College Station, Texas, United States

**Keywords:** Monte Carlo, diabetes, remote health, biosensors

## Abstract

**Significance:**

Continuous glucose monitors (CGMs) are increasingly utilized as a way to provide healthcare to the over 10% of Americans that have diabetes. Fully insertable and optically transduced biosensors are poised to further improve CGMs by extending the device lifetime and reducing cost. However, optical modeling of light propagation in tissue is necessary to ascertain device performance.

**Aim:**

Monte Carlo modeling of photon transport through tissue was used to assess the luminescent output of a fully insertable glucose biosensor that uses a multimodal Förster resonance energy transfer competitive binding assay and a phosphorescence lifetime decay enzymatic assay.

**Approach:**

A Monte Carlo simulation framework of biosensor luminescence and tissue autofluorescence was built using MCmatlab. Simulations were first validated against previous research and then applied to predict the response of a biosensor in development.

**Results:**

Our results suggest that a diode within the safety standards for light illumination on the skin, with far-red excitation, allows the luminescent biosensor to yield emission strong enough to be detectable by a common photodiode.

**Conclusions:**

The computational model showed that the expected fluorescent power output of a near-infrared light actuated barcode was five orders of magnitude greater than a visible spectrum excited counterpart biosensor.

## 1 Introduction

Since the first indwelling medical device, a pacemaker in the 1960s, at least 8% to 10% of Americans have utilized an indwelling medical device in their healthcare.^1^ In recent years, research toward the design and development of indwelling sensors has increased due to their potential to detect near real-time physiological parameters for monitoring chronic diseases, such as cancer, cardiovascular disease, gout, and diabetes.^2^^–^^5^ In particular, indwelling biosensors used for monitoring the glucose concentration in patients with diabetes are among the most heavily researched due to the increasing prevalence of diabetes and the deleterious comorbidities that accompany insufficient monitoring of the condition.^6^^,^^7^ According to the Center for Disease Control 2020 National Diabetes Statistics Report, as of 2018, diabetes is estimated to directly affect over 34 million American adults (7.3 million undiagnosed) with an additional 88 million American adults characterized as prediabetic.^8^ The current focus for indwelling sensors in diabetes has been to mitigate deleterious effects and improve the quality of life by obtaining near real-time glucose data to help the patient and health care provider manage diabetes.^7^^,^^9^

Several researchers have been exploring a grain-of-rice-sized insertable glucose biosensor that converts a chemical signal into an optical signal through a combined recognition component [e.g., competitive binding molecule or glucose oxidase (GOX)] and transduction component [e.g., Förster resonance energy transfer (FRET) or phosphorescence lifetime].^10^^–^^14^ This is unlike currently available commercial continuous glucose meters such as the transcutaneous sensors Dexcom G6 and Abbott Freestyle, as well as the fully implantable Eversense by Senseonics. The commercial transcutaneous continuous glucose monitor are relatively expensive, and since they are transcutaneous, they open the patient to infection and last only 2 weeks primarily due to the eventual loss of skin adhesive. The Eversense was the first fully implantable glucose biosensor, but the transduction assay and optoelectronics are housed in a relatively large capsule, which is implanted by a physician and subsequently transmits data to an external wearable.

A biosensor that includes only the luminescent assay can overcome the limitations of these current devices by being smaller and hence insertable via hypodermic needle—eliminating the need for a physician to implant/explant the biosensor—as well as with potentially better biocompatibility and better cost-effectiveness. However, such an indwelling optically functionalized device mandates that a device external to the body provide excitation light that travels through the skin to the implant and generates a response with a great enough intensity to be read by a photodiode on the skin’s surface. The scattering and absorptive nature of skin, along with endogenous fluorescence, makes this a difficult task.^15^^,^^16^ Additionally, features such as skin tone or body mass index can vary across populations, making optical signal transduction more difficult and highly variable.^17^ However, tools such as Monte Carlo simulation can be used to predict autofluorescence, scattering, and absorptive events. Thus, it can be used to aid device design to overcome these potential barriers.

Monte Carlo modeling is a frequently used computational method for predicting seemingly nondeterministic situations governed by a probability distribution and multiple degrees of freedom. Monte Carlo modeling is used regularly in engineering, basic sciences, and business, among others.^18^^–^^20^ Wang et al.^21^ developed a Monte Carlo model of steady-state light in multilayered tissues, a tool that has been used extensively for modeling light transport through layered tissues. This tool not only has a strongly supported design of optical systems intended for interaction with biological tissue but also has spawned a large number of subsequent Monte Carlo programs for varying applications. Such tools have been used to analyze Raman spectroscopy, polarized light, and birefringent media.^22^^–^^24^ It is also frequently utilized within the device commercialization process for either Food and Drug Administration approval or device optimization, as was the case with Phillips’ BiliCheck.^25^

As a stochastic numerical method, Monte Carlo for photon transport utilizes solutions to the radiative transfer equation informed by materials properties (most commonly absorption coefficient, $\mu_{a}$; scattering coefficient, $\mu_{s}$; anisotropy, g; refractive index, n) of media within a domain to determine photon paths. Briefly, a photon is assigned a given “weight” and deposits that weight as it undergoes absorption events or is scattered by turbid media. These interactions occur at a step size informed by the media being traversed and occur at media boundaries.^26^ More recently developed Monte Carlo programs for photon transport enable simulation of increasingly complex scenarios through the inclusion of tissue autofluorescence, luminescent implants, and inhomogeneous geometries in the X, Y, and Z axes. Furthermore, computational advancements such as graphics processing unit (GPU) parallelization have enabled increasingly complex simulations to be realized.^27^^–^^31^ This work is such an example, as the simulation domain is fluorescent and phosphorescent and represents a discrete insertable biosensor.

Here, we present the development and validation of a Monte Carlo computational framework that analyzes the fluorescent and phosphorescent performance of a grain-of-rice, fully insertable “barcode” glucose biosensor with the goal of overcoming the aforementioned limitations of optically functionalized and indwelling biosensors. To the author’s best knowledge, there are no references in existing literature referring to a multimodal and fully insertable optical glucose biosensor, and there does not exist a Monte Carlo code framework to evaluate the spectral output of FRET assays in bulk tissue. First, the framework is developed and validated against glucose biosensing assays that are already developed and in the literature and are excitable with a wavelength of 450 nm.^32^^,^^33^ Next, the model is extended to analyze the performance of an in-progress device with an excitation wavelength of 680 nm, as red light penetrates tissue more successfully, particularly for darker skin tones.^34^^,^^35^ We aim to develop a computational workflow that can be used to assess phosphorescence-based and FRET-based insertable biosensors, as well as to determine whether in-development assays will yield sufficient photoluminescent signal to be detectable by an off-the-shelf photodiode.

## 2 Materials and Methods

### 2.1 Anatomical Model, Optical Properties, and Computation

**Fig. 1:**
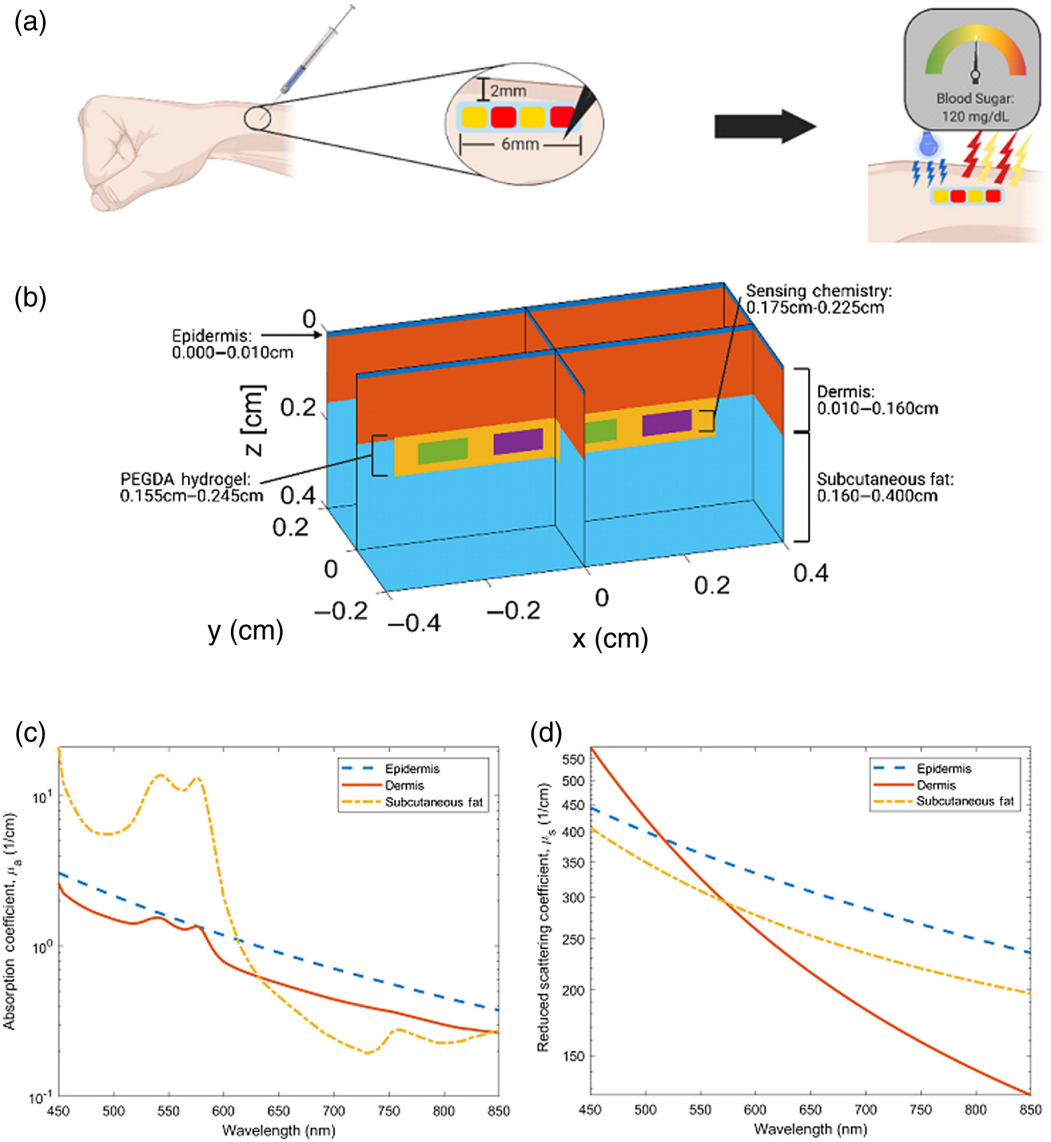
Model anatomy, insertable geometry, and optical properties: (a) dorsal forearm anatomy with proposed bar-code insertable, (b) model geometry with implant, (c) absorption coefficient of tissues, and (d) scattering coefficient of tissue. (a) Created with biorender.com

A 3D model representing the dorsal forearm was chosen as the model anatomy and is given viewed in Fig 1(a). Three anatomical layers were used, and the insertable biosensor was centered at a depth of 0.20 cm. Figure 1(b) provides thicknesses for each element. An epidermal thickness of 0.010 cm was chosen due to literature values ranging between 0.0075 and 0.012 cm.^36^^,^^37^ A dermal thickness of 0.15 cm was used along with a 0.24-cm thick layer of subcutaneous fat.^38^ Figures 1(c) and 1(d) depict the absorption and reduced scattering coefficients used in all simulations, unless otherwise stated. The properties used in these simulations were largely native to MCmatlab and were derived from Jacques et al., with the exception of the epidermal absorption coefficient (which was changed to 1/10th of the native value to reflect the literature).^15^^,^^39^ Refractive index matching was used, and anisotropy was set to g=0.90.

For this work, the source and detector are colocated to eliminate any spectral effects caused by source/detector spacing that may lead to misrepresentation of emission spectra. The source is a rectangular LED-type emitter with a top-hat near field and Lambertian far-field distribution. There is a beamwidth of 0.40 cm in the x-direction and 0.40 cm width in the y-direction. This yields a total illumination area of $0.16\;{\mathrm{cm}}^{2}$ and was chosen to approximate the illumination area of the central green LED on the Apple Watch. In the far-field, a Lambertian distribution with a width of π/8  rad in the x and y directions was used. The detector is a fiber with a numerical aperture of 0.4 and a diameter of 0.17 cm, located at x=0, y=0, and z=0. This diameter was chosen to mimic the sensing area of the SFH 2704 Photodiode (OSRAM Opto Semiconductors). All simulations were performed on a Lenovo Legion Y720 Laptop. GPU-parallelized simulations were completed on a GeForce GTX 1060. The simulation time varied from $10^{7}$ to $10^{8}\text{ photons}/\min$. These values are determined by the overall absorption and scattering of the simulation iteration, as a simulation with a less turbid domain (lower absorption and scattering coefficients) would lead to more interactions before the photon weight reached zero. Simulation time was dependent on the number of interactions was dependent on the number of interactions being calculated as well as the optical properties of the simulation domain and varied from $10^{7}$ to $10^{8}\text{ photons}/\min$. Overall, it was found that a complete iteration of the computational modeling, which would yield the emission spectra of all assays within the barcode biosensor, would take between 2 and 3 h.

Fluorescence models were completed by simulating excitation light coming from the source and reaching the biosensor, and then fluorescence simulations were run for the emission spectra of a given fluorophore using a modified quantum yield (mΦ). This quantum yield was determined by the expression

$$\Phi *\frac{\Phi_{\lambda }}{\int Emd\lambda }=m\Phi .$$ (1)

This was completed to ensure that results were calculated with the correct tissue optical properties at a given wavelength. The quantum yield of a fluorophore (Φ) was multiplied by the ratio of the relative quantum yield at a given wavelength ($\Phi_{\lambda }$) divided by the sum of relative quantum yields for each relevant wavelength (*Em* = peak-normalized emission spectra). For input into MCmatlab, mΦ was further modified by multiplying by the ratio of excitation wavelength to emission wavelength, as MCmatlab accepts power yield.

Each dye emission spectra was simulated via 80 individual emission simulations, yielding a resolution between 1.8 and 2.6 nm depending on the width of the spectrum. Thus, a given assay will have a single excitation simulation, 80 emission simulations for phosphors, 80 simulations for the FRET donor dye, and 80 simulations for the FRET acceptor dye. Note S2 in the Supplementary Material details the number of simulations required to determine the output of a barcode biosensor iteration. After all simulations were completed, postprocessing was conducted to calculate time-resolved results for phosphorescent fluorophores and the final emission spectra of FRET species.

### 2.2 Visible Spectrum Biosensor

**Fig. 2:**
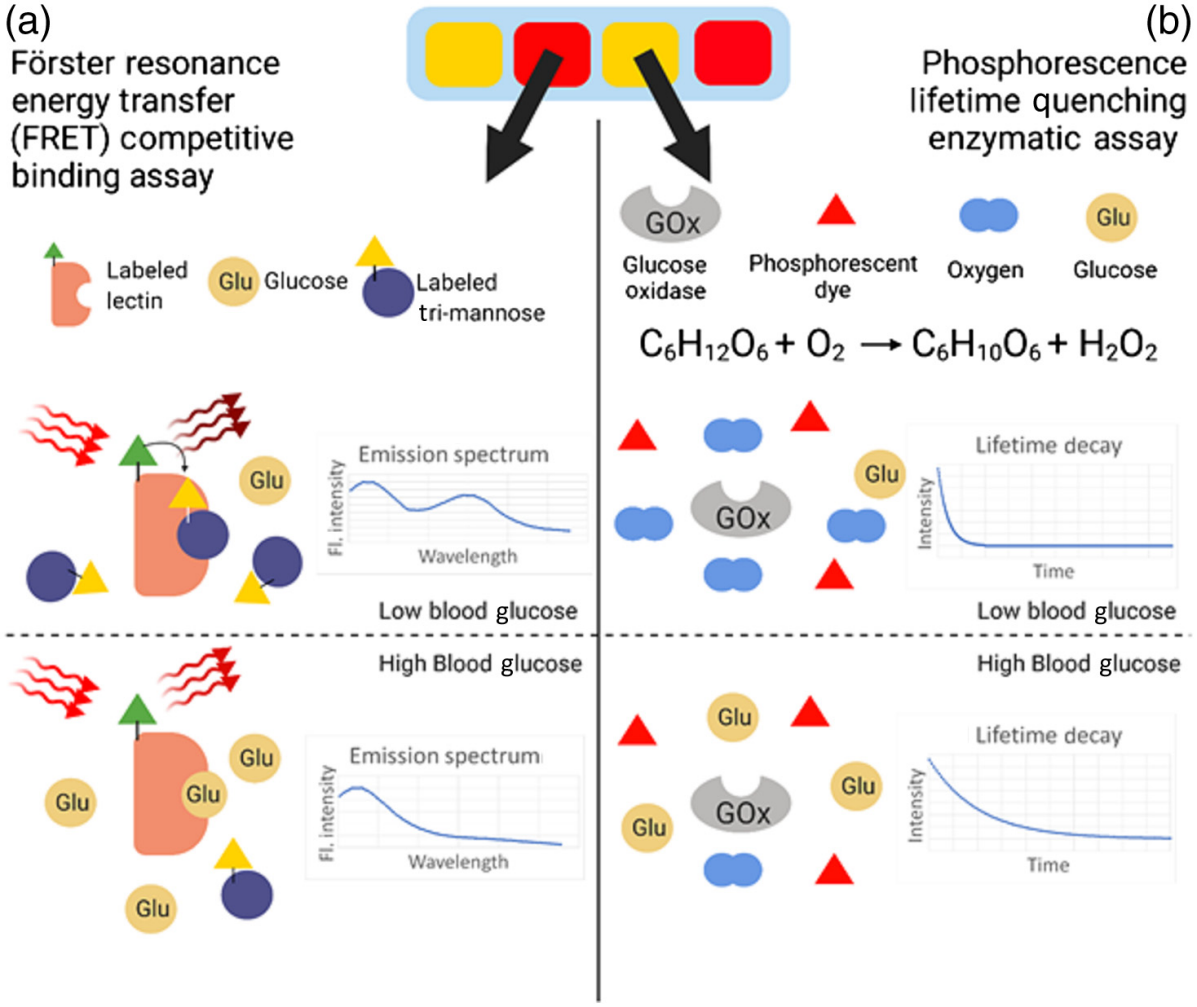
(a) FRET competitive binding assay and (b) phosphorescence lifetime quenching enzymatic assay.

A decay modality for glucose sensing was reported previously by Brown and McShane^12^ wherein the oxidation of glucose, catalyzed by the enzyme GOX, resulted in a decreasing concentration of molecular oxygen within a hydrogel. This decreasing concentration was then correlated to the phosphorescence lifetime of a dye also embedded in the hydrogel matrix, as oxygen is a strong triplet-state quenching agent.^12^^,^^40^^,^^41^ This is depicted in Fig. 2(b). Although a singlet state fluorophore may have a lifetime on the order of $10^{-9}\;s$, the phosphors used in this assay have lifetimes on the order of $10^{-4}\;s$. The use of time-domain phosphorescent lifetime measurements is beneficial, as skin autofluorescence exhibits the short lifetimes that accompany singlet state excitation and thus do not interfere with the longer lifetimes associated with triplet state excitation if the emission acquisition is properly gated.^42^^–^^44^ In this model, the domains containing this assay [represented as green regions in Fig. 1(a)] have an absorption coefficient derived from the extinction coefficient of the phosphorescent dye, Pd-meso-tetra(4-carbodyphenyl)porphyrin (PdP), determined as

$$\mu_{a}=2.303*\varepsilon *[C],$$ (2)

where ε is the extinction coefficient and [C] is the molarity of the species. Scattering for this domain is assumed to be negligible, given that the absorption coefficient is multiple orders of magnitude greater than the scattering coefficient. After simulations are completed and a phosphorescence emission spectrum is obtained, the Stern–Volmer (SV) equation

$$\frac{I_{0}}{\Delta I}=\frac{\tau_{0}}{\Delta \tau }=1+K_{sv}*[Q],$$ (3)

is then applied using phosphorescence intensity and a user-inputted oxygen concentration to determine phosphorescence intensity after quenching. Once all relevant material properties have been collected, a first simulation is conducted again with model geometry that has wavelength-independent optical properties (unlike skin). The emission spectra collected from these simulations were then normalized and compared with published data to validate the modeling framework.^32^^,^^33^ Once agreement was determined to be sufficient, simulations of the biosensor were completed with a geometry and model properties representative of tissue, and iterative simulations were completed to identify differences in response between turbid and nonturbid environments.

The second sensing modality was previously reported by Meadows and Schultz^45^ and improved upon by Cummins et al.^46^ Using FRET, the competitive binding between substrates glucose and dye-bound mannatetraose (APTS-MT) to enzyme Concanavalin A (ConA), which is bound to tetramethylrhodamine isothiocyanate (TRITC), is represented by the ratio of two fluorescent emission peaks as the donor dye (APTS) engages in efficient long-distance dipole–dipole coupling with the acceptor dye (TRITC) when occupying the active site of ConA. As glucose concentration increases, the acceptor peak decreases in relative intensity because energy transfer is reduced when the competing ligand is displaced from the ConA as glucose occupies more active sites. This is depicted in Fig. 2(a). The absorption coefficient is derived from the donor, which absorbs the excitation light. Fluorescence simulations were completed for the donor and acceptor separately to identify the effect of tissue-like media on emission spectra. The final combined emission spectra for this assay were determined as

$$\frac{{\mathrm{FI}}_{\text{Donor}}}{\mathrm{SI}}={\mathrm{FLRD}}_{\text{Donor}}*(1-\mathrm{SO}*E*\mathrm{ConA}*\mathrm{MT}*\kappa 2),$$ (4)

and

$$\frac{{\mathrm{FI}}_{\text{Acceptor}}}{\mathrm{SI}}={\mathrm{FLRD}}_{\text{Acceptor}}*\;\frac{\varepsilon_{D}}{\varepsilon_{A}}\;*\mathrm{SO}*E*\mathrm{ConA}*\kappa^{2},$$ (5)

for the donor and acceptor, respectively, and then were additively combined. To determine the fluorescent intensity as a fraction of source intensity ($\frac{{\mathrm{FI}}_{\text{Acceptor }\mathrm{OR}\text{ donor}}}{\mathrm{SI}}$), the fluorescent light reaching the detector (FLRD) was modified by parameters determined via the binding kinetics, such as the percent of bound Concanavalin A (ConA) and Mannotetraose (MT); the fluorophore excitation and emission spectra, such as the spectral overlap (SO); the FRET efficiency (E), the relative extinction coefficient of each fluorophore $\left(\varepsilon_{D},\varepsilon_{A}\right)$; and the orientation factor, $\kappa^{2}$. To validate this component of the framework, the emission spectra of two combinations of the assay, representing high and low glucose concentrations, were simulated with the framework and then compared with experimental fluorescence data of free-solution versions of the assay with identical concentrations: 1  μM TRITC-ConA: 100 nM APTS-MT and 500 nm TRITC-ConA: 100 nM APTS-MT.^13^ Good agreement between the data generated by the computational framework and data found in previous literature indicates that the model can accurately determine the fluorescent response of this FRET assay.

Simulations and postprocessing were completed using optical properties representing wavelength-independent media, as described previously, to compare with published literature and ensure accuracy at the assay-specific ratiometric values of 520 and 600 nm. Then, simulations were completed in tissue to identify any changes in spectra associated with the turbidity of skin.

### 2.3 Tissue Autofluorescence

**Fig. 3:**
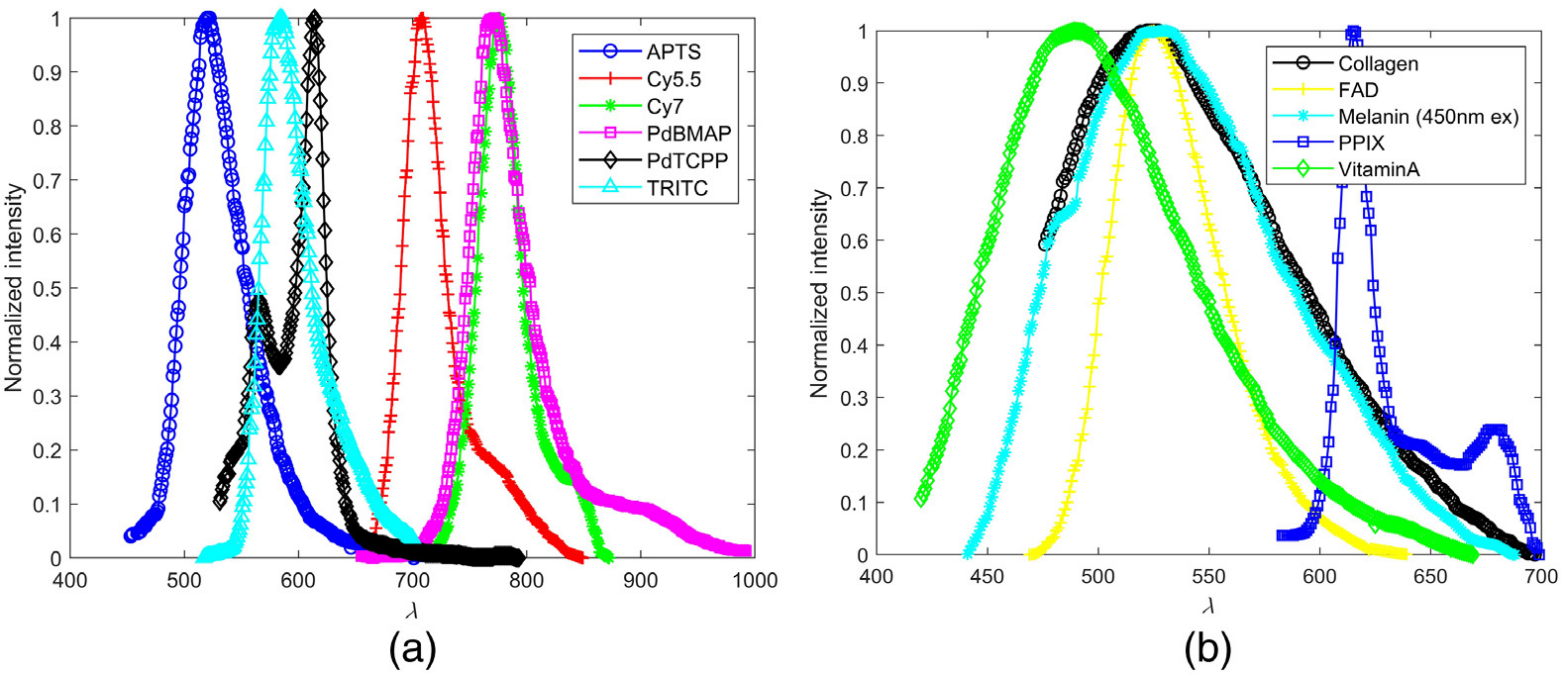
(a) Dye emission spectra and (b) endogenous fluorophore emission spectra.^33^^,^^48^^–^^54^

An additional set of models was used to replicate autofluorescence, fluorescence light emitted by endogenous fluorophores within the tissue, at excitation wavelengths of 450 and 680 nm. This was done to analyze spectral characteristics of fluorescence originating from fluorophores of interest, namely, flavin adenine dinucleotide (FAD), collagen, melanin, and vitamin A. The common endogenous fluorophore nicotinamide adenine dinucleotide was excluded because it is not excited with 450-nm light.^47^ The resultant spectra were compared with the fluorescence output of the biosensor. The spectral properties of these fluorophores are illustrated in Fig. 3(b).

A different model was created for each individual endogenous fluorophore. All models have a three-layer base geometry (epidermis/dermis/subcutis). The optical properties of the layer for which a given endogenous fluorophore resides were modified to represent fluorescence as determined by values from the literature. As shown in Table 1, the absorption coefficient and quantum yield were modified. It was then assumed that all absorption that occurred was completed by endogenous fluorophores. The FAD layer was in the epidermis (excluding the stratum corneum, which occupied the top 0.20 mm), the melanin layer was in the stratum basale, and collagen and vitamin A occupied the dermis.^62^^,^^78^^–^^80^ Equation (2) was used to determine the absorption coefficient of a fluorophore layer, the reduced scattering coefficient remained constant from the original layer, and quantum yield was derived from the literature. Simulations were completed at a resolution ≤5  nm.

**Table 1:** Fluorophore properties.

Fluorophore	Excitation wavelength (nm)	$\mu_{a}$ (${\mathrm{cm}}^{-1}$)	Φ
PdP	450	0.0066^32^^,^^55^^,^^56^	0.068^48^^,^^57^^,^^58^
TRITC-ConA	450	0.001716^1^^,^^33^^,^^51^	0.1^59^
APTS-MT	450	n/a	0.2
Collagen	450	7.8131^60^^,^^61^	0.05
Vitamin A	450	0.0207^62^^,^^63^	0.03^64^
FAD	450	0.3643^65^	0.033^66^
PPIX	450	0.1058	0.085^67^
Melanin	450/680	14.24/1.03^68^^,^^69^	0.0005/0.00056^70^
Cy5.5-MT	680	250.0^58^^,^^71^	0.2^72^
Cy7-ConA	680	n/a	0.3^73^^–^^75^
BMAP	680	168.0^5^^,^^75^^–^^77^	0.21^48^^,^^76^

Melanin autofluorescence with an excitation wavelength of 680 nm was also simulated. The emission spectrum and quantum yield at this excitation wavelength could not be found in published literature; therefore, it was determined experimentally via published protocols^70^^,^^81^ using a PTI Quantamaster series fluorescence spectrometer and a Cary 300 Series UV-Vis Spectrophotometer, Agilent Technologies. The linear relationship comparing the absorbance and fluorescence of five solutions of synthetic eumelanin (Melanin, Sigma Aldrich) dissolved in ammonium hydroxide (0.033, 0.027, 0.02, 0.013, and 0.0067 mM) was compared to the same parameter from a known standard, Alexafluor-680 (AF-680, Thermo Fisher Scientific, Massachusetts) dissolved in phosphate-buffered saline (PBS). The quantum yield of melanin was then calculated according to

$$\Phi_{\mathrm{Mel}}=\Phi_{\mathrm{AF}-680}\frac{{\text{Gradient}}_{\text{Melanin}}}{{\text{Gradient}}_{\mathrm{AF}-680}}*\frac{{\left(n_{\text{Melanin}}\right)}^{2}}{(n_{\mathrm{AF}-680}{)}^{2}},$$ (6)

where “Gradient” is the slope of the best fit line between absorbance and fluorescence and n is the refractive index.

### 2.4 Far-Red Biosensor

A phosphorescence lifetime decay modality described previously was simulated in this work.^44^ This domain works identically to the visible spectrum counterpart but utilizes the phosphorescent dye Palladium (II) tetramethacrylated benzoporphyrin (BMAP) and an excitation wavelength of 680 nm. Similarly, a far-red FRET assay is modeled using Cy5.5 and Cy7 as its donor and acceptor, respectively, according to Eqs. (4) and (5). The simulated fluorescence of the far-red FRET assay differs from the visible spectrum counterpart in that both the donor and acceptor dyes emit fluorescence when excited with 680 nm. Thus, simulating the fluorescent output of the far-red FRET assay consisted of two components: simulation of fluorescence originating from FRET with the assumption that the binding kinetics of APTS-MT and TRITC-ConA will be similar to Cy5.5-MT and Cy7-ConA and an additional computation of Cy7 fluorescence emission with a quantum yield of 0.30 and an absorption coefficient of $42.5\;{\mathrm{cm}}^{-1}$. The latter computation was completed by changing the quantum yield and absorption coefficient to the previously mentioned values and not applying Eqs. (4) and (5) in postprocessing. The results of both simulations were then additively combined to generate the final result.

### 2.5 Power Analysis

Finally, the photoluminescent output of the visible and far-red assays (four in total) was compared with the detectability limits of the SFH 2704 (OSRAM Opto Semiconductors), a photodiode with a wide sensing window that has been applied to healthcare applications.^82^^,^^83^ The minimum power detectable by the selected photodiode was calculated via

$$P_{\lambda ,\min}=\mathrm{NEP}*{\mathrm{BW}}^{\frac{1}{2}}*\frac{S_{\lambda }}{S_{\max}},$$ (7)

where P is the power, NEP is the noise equivalent power, BW is the bandwidth, and S is the spectral sensitivity. To compare output power from the Monte Carlo simulations with the minimum power detectable by the SFH 2704, an incident power of the excitation source was assigned using the maximum permissible exposure of skin to illumination from light of wavelengths 450 and 680 nm. This was found to be 32 mW or $2\;\mathrm{kW}/m^{2}$ for an LED with an area of $0.16\;{\mathrm{cm}}^{2}$ for both excitation wavelengths.^84^

### 2.6 Parametric Analysis for Model Convergence

A parametric convergence study was completed to determine the number of photons required to accurately represent biosensor excitation and emission such that the result of a simulation with $10^{x\;}\mathrm{photons}$ was <5% different than the result of a simulation with $10^{x+1}\text{ photons}$. For these simulations, the model parameters described in Fig. 1 were used, except for the absorption coefficient and quantum yield of the target fluorophore. The target fluorophore was assigned the low absorption coefficient and power yield of $0.001\;{\mathrm{cm}}^{-1}$ and 0.001 (defined as the quantum yield multiplied by the ratio of emission wavelength to excitation wavelength), respectively. This was held constant over the emission spectrum.

These parametric convergence study simulations were conducted with an excitation of 450 (emission: 493 to 700 nm) and 680 nm (emission: 700 to 900 nm) to match the excitation and emission wavelengths used in this work, and the results are viewable in Fig. S1 in the Supplementary Material and described in Note S1 in the Supplementary Material. Simulations were repeated with an increasing number of photons until it was observed that simulations with $10^{7}\text{ photons}$ yielded results <5% different in magnitude of light reaching the photodetector when compared with simulations completed with $10^{8}\text{ photons}$. It was found that this also maintained the coefficient of variation (COV) below 1%. Thus, $10^{7}\text{ photons}$ were used for all simulations in this work.

### 2.7 Parametric Sweep for Implant Depth and Volume Fraction Melanosomes

Parametric sweeps were conducted to determine the decrease in optical power associated with increasing the implant depth and increasing the amount of melanin in the epidermal layer. To quantify the effect of implant depth, the depth of the center of the biosensor was increased from 0.20 to 0.50 mm, and simulations were completed in triplicate. To quantify the effect of volume fraction of melanosomes, three values of 0.03, 0.23, and 0.43 were chosen as representative of individuals with Fitzpatrick skin tone I, Fitzpatrick skin tone IV, and Fitzpatrick skin tone VI.^69^ Then, epidermal optical properties were recalculated to be representative of increased melanin concentration.^15^

### 2.8 Summary of Methods

In summary, this computational framework seeks to predict the optical power output of a “barcode” glucose biosensor in three parts, as shown in Table 2. First, MC models of existing phosphorescence lifetime decay and FRET assays with an excitation wavelength of 450 nm are created and validated against results presented in previous literature that demonstrate the glucose-sensing abilities of these assays in an *in vitro* setting. The phosphorescence lifetime decay assay is validated by comparing emission spectrum and emission intensity over time (lifetime). The FRET assay is validated by comparing the emission spectrum over high and low physiological glucose concentrations. Second, tissue autofluorescence is simulated by analyzing the fluorescence emission from significant endogenous fluorophores in tissue, and the subsequent model is validated by comparing the computationally generated emission spectrum with a reference spectrum from the literature. Lastly, the computational capabilities developed in the prior two components are applied to predict the photoluminescent output of a “barcode” biosensor with an excitation wavelength of 680 nm, as well as the associated autofluorescence emission.

**Table 2:** Summary of methods.

Workflow component	Description	Notes
Existing biosensor assays (450-nm excitation)	PdP-based phosphorescence lifetime decay	Replication of work in Ref. 32
	APTS-MT/TRITC-ConA FRET assay	Replication of work in Ref. 33
Tissue autofluorescence	450-nm excitation tissue autofluorescence	Replication of work in Ref. 16
	680-nm excitation tissue autofluorescence	Application of validated tissue autofluorescence simulation
Far-red barcode biosensor (680-nm excitation)	BMAP-based phosphorescence lifetime decay	Application of validated phosphorescence lifetime decay assay
	Cy5.5-MT/Cy7-ConA FRET assay	Application of validated FRET simulation

## 3 Results and Discussion

### 3.1 Visible Spectrum Barcode

**Fig. 4:**
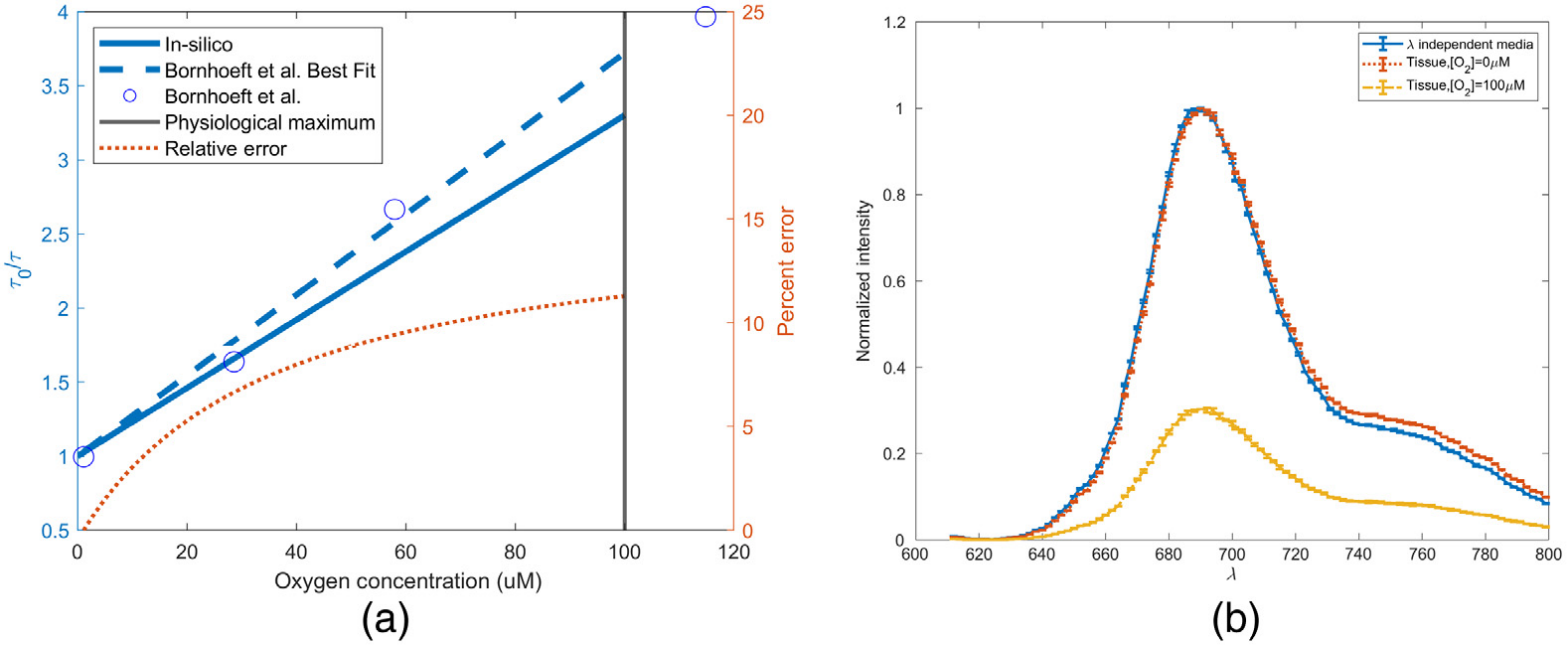
(a) SV for validation of *in silico* PdTCPP time-resolved measurement and (b) change in emission spectra as a function of tissue.

Palladium meso-tetra(4-carboxyphenyl)porphyrin (PdTCPP) based sensing chemistry was simulated *in silico* in nonwavelength-dependent media, and the time-resolved simulation was validated against previous literature using the SV relationship. In Fig. 4(a), the SV response of the sensor found in previous literature (SV constant of $0.023\;{\mu M}^{-1}*$ [$O_{2}$] and a natural lifetime of 588  μs) was compared with the simulated results. Overall, the mean average percent error (MAPE) from 0 mM to the expected operational maximum (100  μM) was found to be 7.87%.^32^^,^^85^ This error is possibly caused by the changing fraction of fluorophore able to be quenched that occurs as a function of the sensing chemistry surface area. The SV relationship applies not only to lifetime but also to spectral intensity. Figure 4(b) illustrates the decrease in intensity that is associated with the physiological maximum oxygen concentration, as well as the spectral shift observed when the simulation was performed with *in vivo* optical properties. A 2-nm redshift is observed after 700 nm and results in the weighted average of the spectra changing from 708 to 710 nm. Additionally, the normalized intensity of a given wavelength after 700 nm is, on average, 2% greater in the wavelength-dependent simulation. This is likely due to the minimal change in tissue optical properties at this wavelength range, compared with that observed between 550 and 600 nm. As shown in Fig. 4(b), peak intensity of the emission spectrum at the physiological maximum oxygen concentration will decrease to ∼30% of the intensity predicted in the absence of oxygen. From this data, we can conclude not only that are we accurately able to replicate the phosphorescence response of this assay, but also that inclusion of such an assay *in vivo* would not require a change in optical filtering because the peak does not change.

**Fig. 5:**
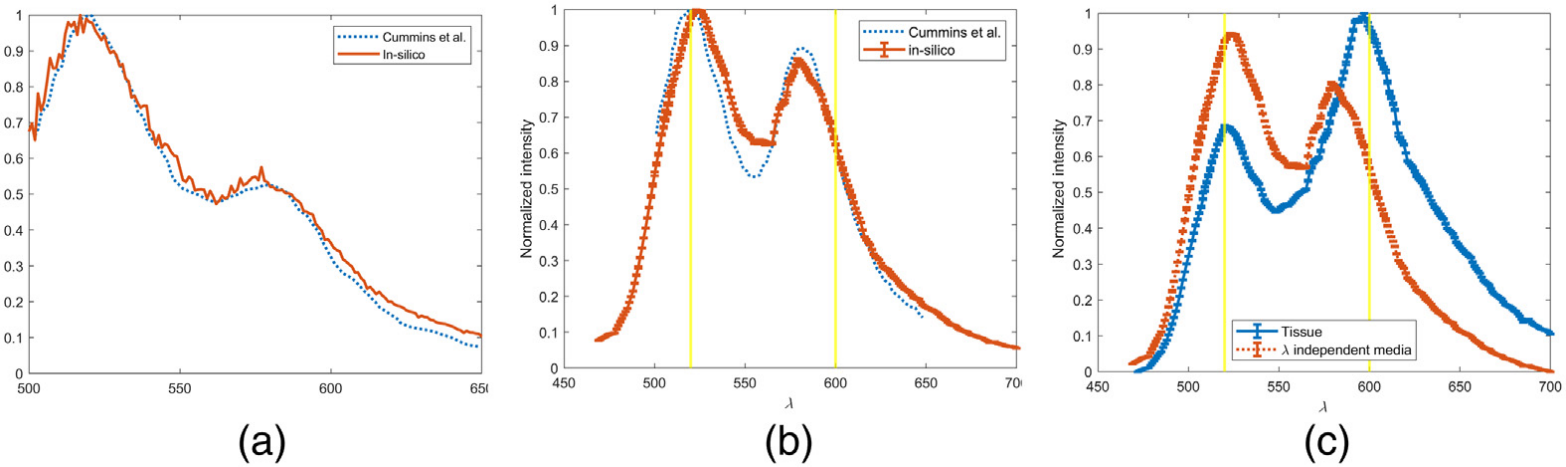
(a) Comparison of simulation and previous literature for 500 nm TRITC-ConA: 100 nM APTS-MT, (b) comparison of simulation and previous literature for 1  μM TRITC-ConA: 100 nM APTS-MT, and (c) simulated change of 1  μM TRITC-ConA: 100 nM APTS-MT spectra *in vivo*.

Figures 5(a) and 5(b) illustrate the comparison of the *in silico* spectra to Cummins.^33^ As a ratiometric assay, glucose concentrations are determined using the relative intensities at 520 and 600 nm. However, matching the entire spectrum provides for a more robust model, allowing for comparisons across the entire emission. Figure 5(a) compares spectra with lower acceptor emission that would be representative of a higher glucose concentration versus that shown in Fig. 5(b), where a more noticeable acceptor peak is observed. Overall, the MAPE between the *in silico* and *in vitro* spectra in Figs. 5(a) and 5(b) was found to be 2.69% and 5.84%, respectively, which is well within a 10% target acceptance. Our model predicts a greater response in the red-end of the emission spectra than has been observed in the literature, but it is accurate in producing the waveform and relative strength of the donor and acceptor fluorophores. Figure 5(c) illustrates the predicted spectral transformation of emission from a 1:10 donor:acceptor ratio when the assay is placed in tissue. A significant shift in spectra is observed. The acceptor peak moves to 597 from 580 nm. This dramatic change is likely caused by the absorptive effects of hemoglobin within the tissue over the same wavelengths. The donor peak also undergoes a blue shift of a smaller magnitude from 523 to 521 nm. This is likely due to the absorption of subcutaneous fat and being higher at 523 than 521 nm (8.4 and $7.8\;{\mathrm{cm}}^{-1}$, respectively), which is in contrast to the general decrease in tissue absorption as wavelength increases. From this data, the created Monte Carlo models demonstrate sufficient agreement with the published data.

**Fig. 6:**
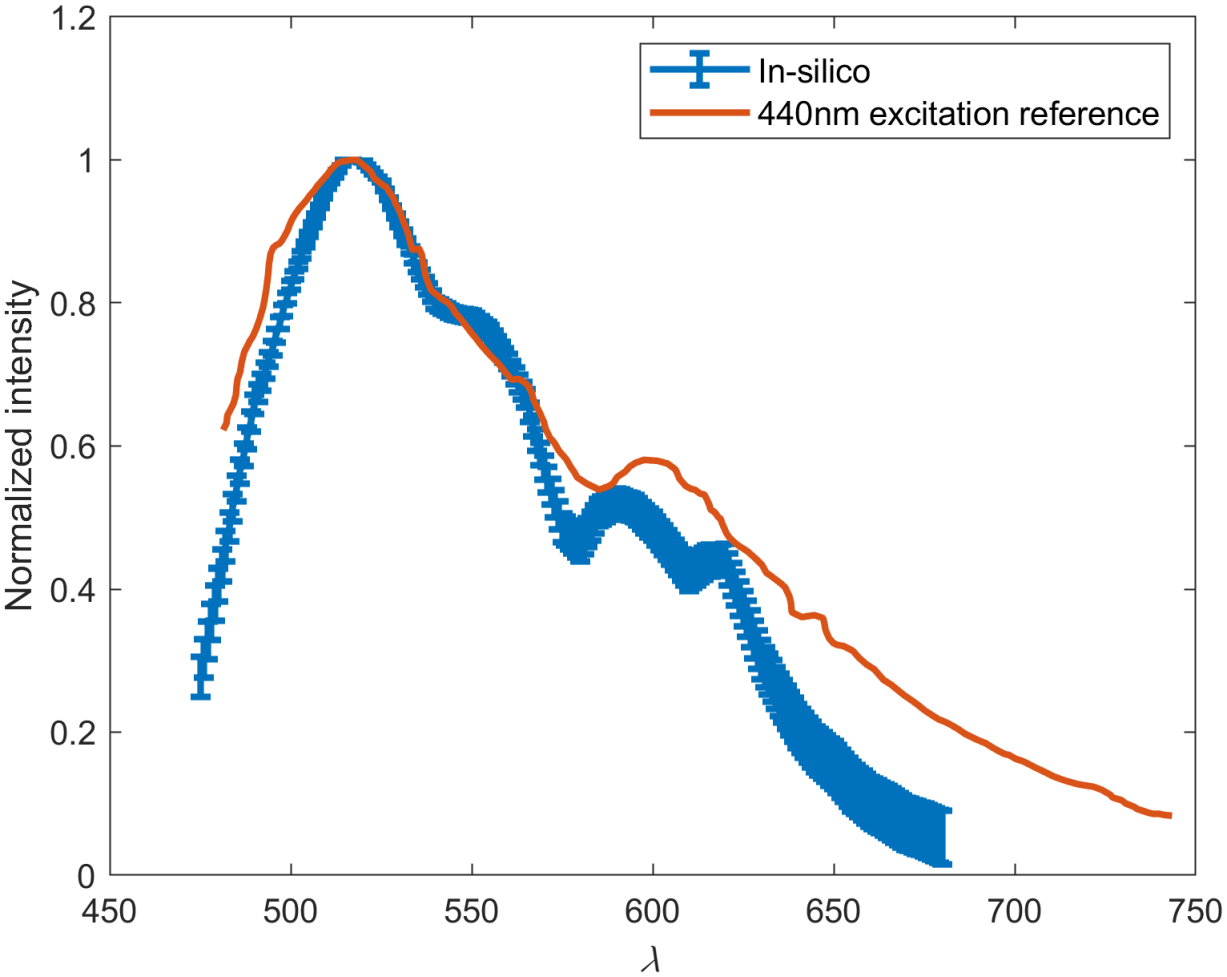
*In silico* 450-nm excitation autofluorescence spectra compared with 440-nm excitation spectra collected by Zeng.^16^

One of the obstacles to implementing insertable fluorescence-based monitoring technology is tissue autofluorescence. Although the phosphorescence decay assay is time-resolved and is able to circumvent endogenous fluorescence via time-gated detection, the ratiometric FRET assay is not. Thus, autofluorescence in tissue at 450 nm was simulated to estimate the effect of endogenous fluorophores on this signal. To ensure a sufficiently high resolution and provide triplicate results of each endogenous fluorophore spectra, a total of 675 simulations of $10^{7}\text{ photons}$ was completed. Figure 6 demonstrates these results as compared with autofluorescence spectra collected from the hand dorsum and inner forearm of Asian and Caucasian volunteers by Zeng et al.^86^ There is a 6.3% difference in simulated spectra compared with the reference data between 482 and 600 nm. However, above 600 nm, this difference increases to 43%. This indicates the model’s ability to determine spectral peaks and its inability to replicate the end of emission spectra. These differences are possibly due to the 10 nm difference in excitation wavelength as well as the lack of specific anatomical data available about the participants used in Zeng et al., specifically dermal thicknesses and melanin concentrations. Most importantly, the simulation confirms a peak at 520 nm and a secondary peak around 600 nm. The primary 520-nm peak is caused by collagen, which is a large source of autofluorescence at most visible spectrum excitation wavelengths and is most important to accurately estimate due to the overlap with the anticipated fluorescence peaks from the FRET assay. Additionally, it is well understood that 450-nm excitation light will not propagate far through tissue. For the phosphorescence assay, only 0.0001% of light from a 450-nm incident LED will be absorbed by the assay. Thus, it is not likely that the 450-nm excitation “barcode” will have sufficient signal compared with the autofluorescence noise determined to exist at these wavelengths. However, the use of Monte Carlo to replicate the spectroscopic and time-resolved assay has been validated and will be used to assess the feasibility of a barcode featuring identical interrogation methods but with a far-red excitation wavelength of 680 nm.

### 3.2 Far-Red Barcode

As mentioned previously, a version of the biosensor with far-red excitation and emission was designed to overcome the limitations of visible light propagation through tissue. This biosensor, consisting of a Palladium (II) tetramethacrylated benzoporphyrin (BMAP) phosphorescence lifetime assay and a Cy5.5/Cy7 FRET competitive binding assay, is anticipated to yield a greater luminescent output with decreased tissue absorption and autofluorescence.

**Fig. 7:**
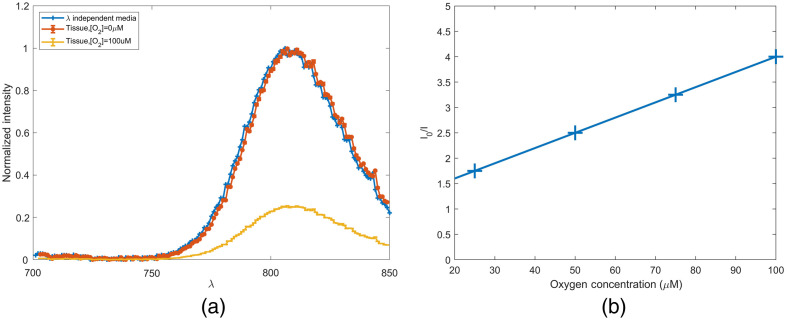
(a) *In silico* 680-nm excitation PdBMAP in wavelength independent media versus tissue and (b) PdBMAP intensity as a function of interstitial oxygen concentration.

The results of simulating a glucose biosensor with 0.91 mM of phosphorescent dye BMAP in wavelength independent media and *in vivo* are shown in Fig. 7(a). Anoxic conditions in wavelength independent media and *in vivo* present nearly identical spectra. A shift of 1 nm is observed, as the *in vivo* spectra peak is located at 807 nm and the wavelength-independent media peak is at 806 nm. This insignificant shift is not anticipated to affect sensor functionality or cause any change to the device design. After determining the effect of physiological maximum oxygen concentration on phosphorescent intensity with an SV coefficient of 0.03, an intensity reduction of 75% is observed in Fig. 7(b).^44^ Although this compares similarly to the 70% loss in intensity for the visible spectrum phosphorescent assay, the near-infrared (NIR) version still performs favorably as it absorbs 5.32% of all excitation light absorbed within the simulation domain. This is four orders of magnitude >0.0001% of incident light absorbed by the visible spectrum assay. This increase can be attributed to the absorption coefficient, which is four orders of magnitude greater than the visible spectrum counterpart, and the increase in light penetration. Tissue irradiance at a depth of 0.2 cm is 68× greater when 680-nm light is used compared with 450-nm light.

**Fig. 8:**
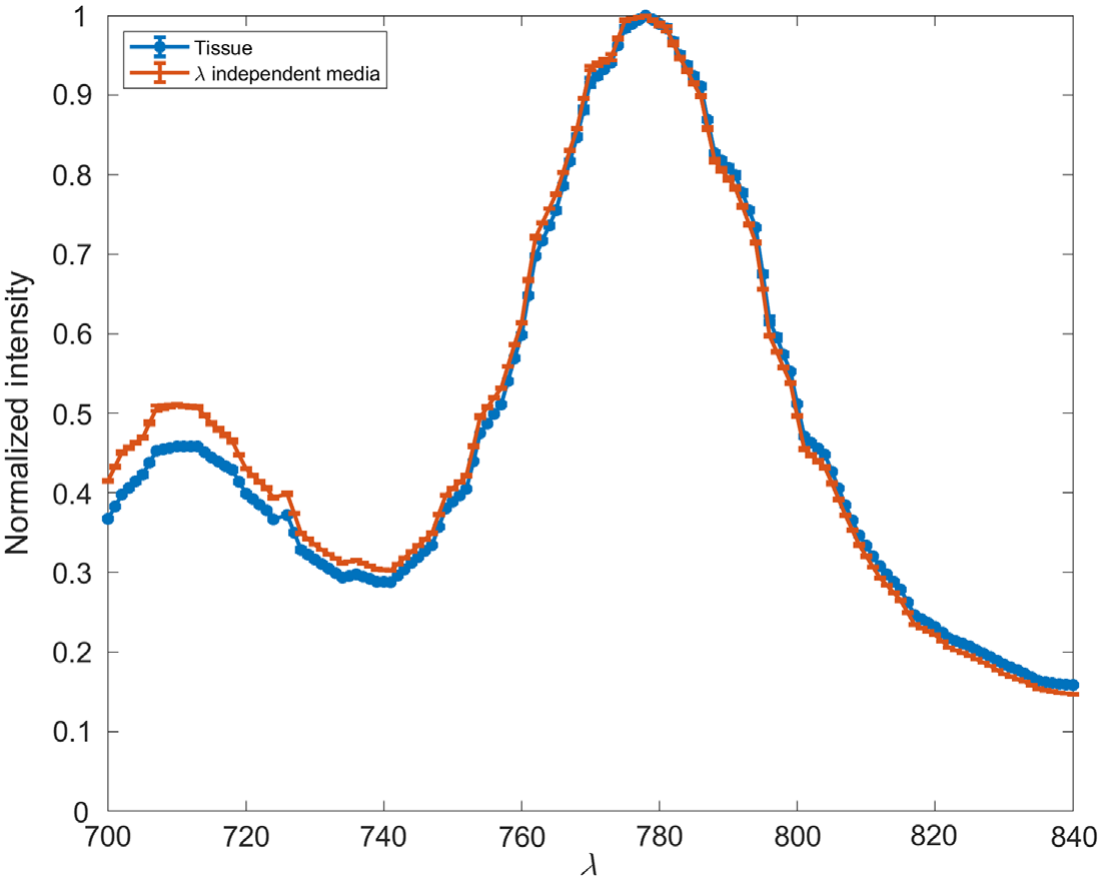
*In silico* 680-nm excitation Cy5.5-ConA and Cy7-MT in wavelength-independent media and tissue.

The far-red version of the FRET assay does not undergo a spectral shift when one compares the wavelength-dependent simulations with wavelength-independent simulations (Fig. 8). However, it does undergo a shift in normalized intensity across the emission spectra. In wavelength-independent media, the ratio of peak intensities (779/710  nm) is 1.96. In wavelength-dependent media, this shifts to 2.18. Across the entire spectra, the weighted mean emission wavelength is 763 nm in wavelength-independent media and 765 nm in skin-like media. This difference, similar to that observed for the phosphorescence lifetime assay, is not significant enough to warrant any consideration as the assay is further developed. The NIR assay performs favorably to the visible spectrum analog, as it absorbs 8.19% of all incident light that was absorbed within the simulation domain, whereas the visible spectrum assay absorbs only 0.000026%.

**Fig. 9:**
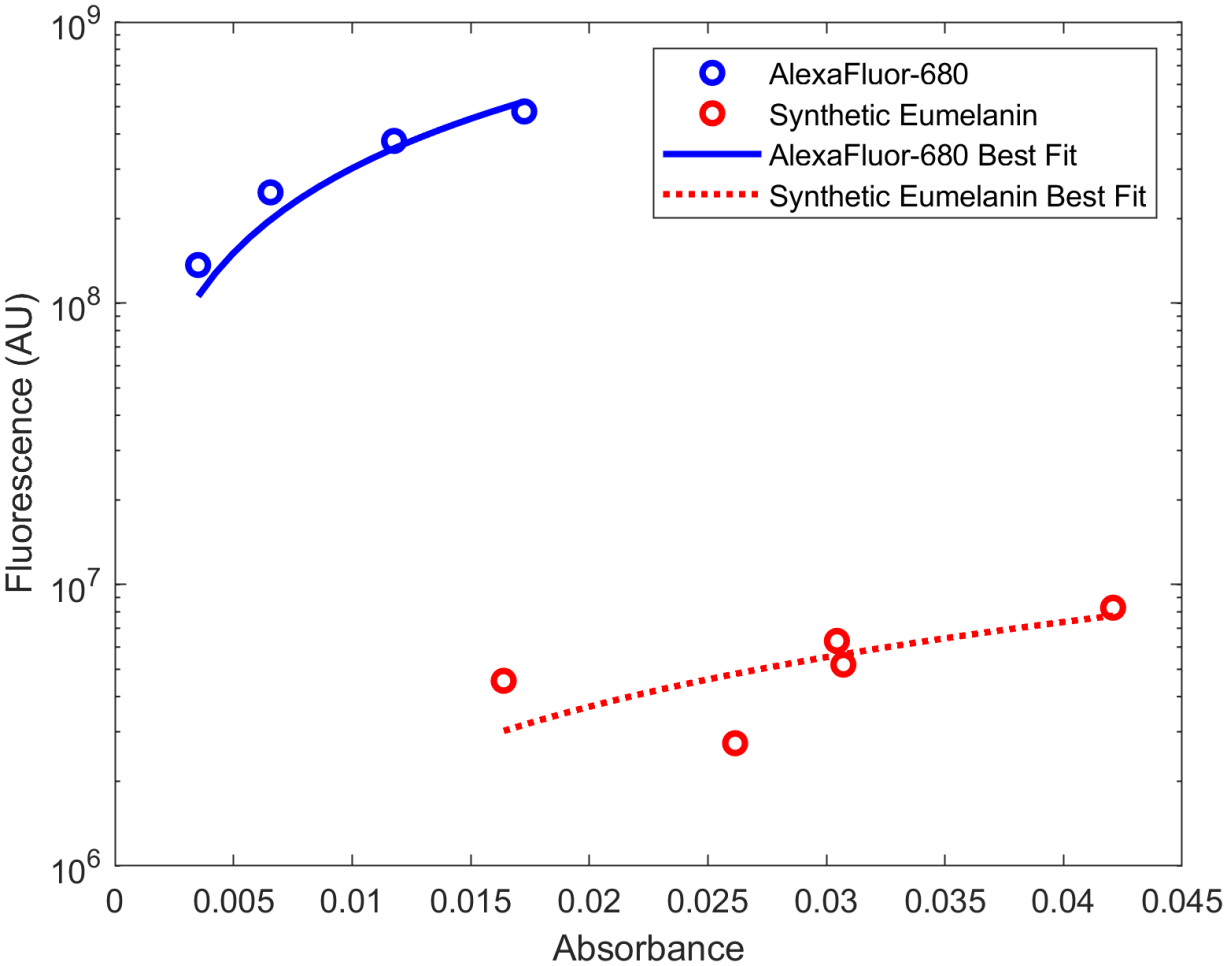
Experimental determination of melanin quantum yield: (blue) AlexaFluor-680 and (red) synthetic eumelanin.

**Fig. 10:**
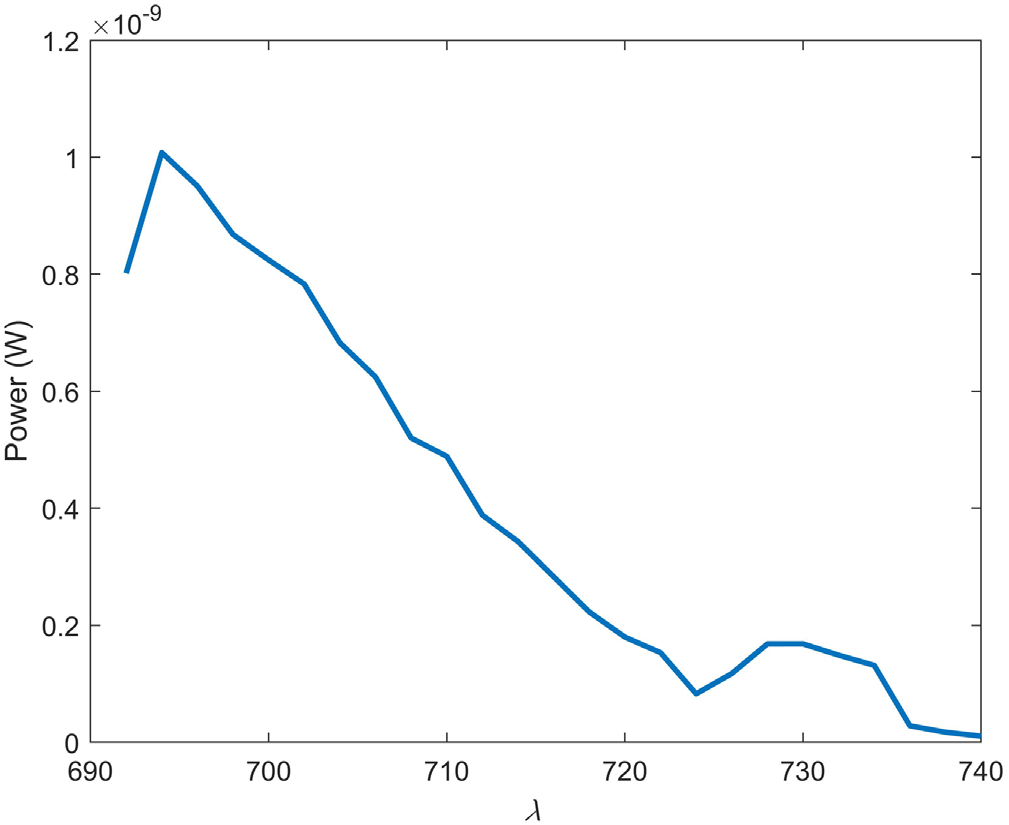
*In silico* 680-nm excitation of melanin in tissue with a 32-mW LED.

As mentioned previously, the 450-nm excitation barcode also induces an autofluorescence spectra that featured a peak overlapping with the ratiometric FRET assay. Thus, it would be prudent to evaluate the endogenous fluorescence expected *in vivo* with an excitation wavelength of 680 nm. Although far-red single-photon endogenous fluorescence has not been studied as thoroughly as visible spectrum autofluorescence, it is well known that far-red autofluorescence is orders of magnitude weaker than its visible spectrum counterpart.^87^^,^^88^ The major endogenous fluorophores in the far-red/NIR range have been reported to be melanin and porphyrins.^53^ However, contradicting literature exists, demonstrating that porphyrins associated with healthy tissue do not excite beyond the red-most Q band, which ends at around 660 nm.^53^^,^^89^ Thus, only melanin was included in the simulations to estimate far-red autofluorescence. First, the quantum yield of melanin at this wavelength had to be determined experimentally by relating melanin fluorescence and absorbance to the same parameters from a known standard. The gradient of synthetic eumelanin in ammonium hydroxide and standard AlexaFluor-680 in PBS is shown in Fig. 9 and was found to be $1.84\times 10^{8}$ and $3.02\times 10^{10}$, respectively. The refractive index of melanin in ammonium hydroxide and Alexafluor-680 in PBS at 680 nm was 1.348 and 1.3284, respectively. The quantum yield of synthetic eumelanin in ammonium hydroxide was then calculated to be 0.00056 according to Eq. (6). The absorption coefficient for an individual with a 3% volume fraction of melanin was set to $4\;{\mathrm{cm}}^{-1}$. As shown in Fig. 10, fluorescence has a peak at ∼695  nm and then decreases with a shoulder at 730 nm. The location of the peak at 695 nm is closest to the 710-nm peak observed for Cy55, and autofluorescence retains ∼50% of its intensity at that wavelength. However, given the relatively narrow 50-nm bandwidth of this spectrum, it is possible that this noise source can be overcome with sufficiently strong emission from fluorophores in the implants.

### 3.3 Power Comparison and Parametric Sweep

**Fig. 11:**
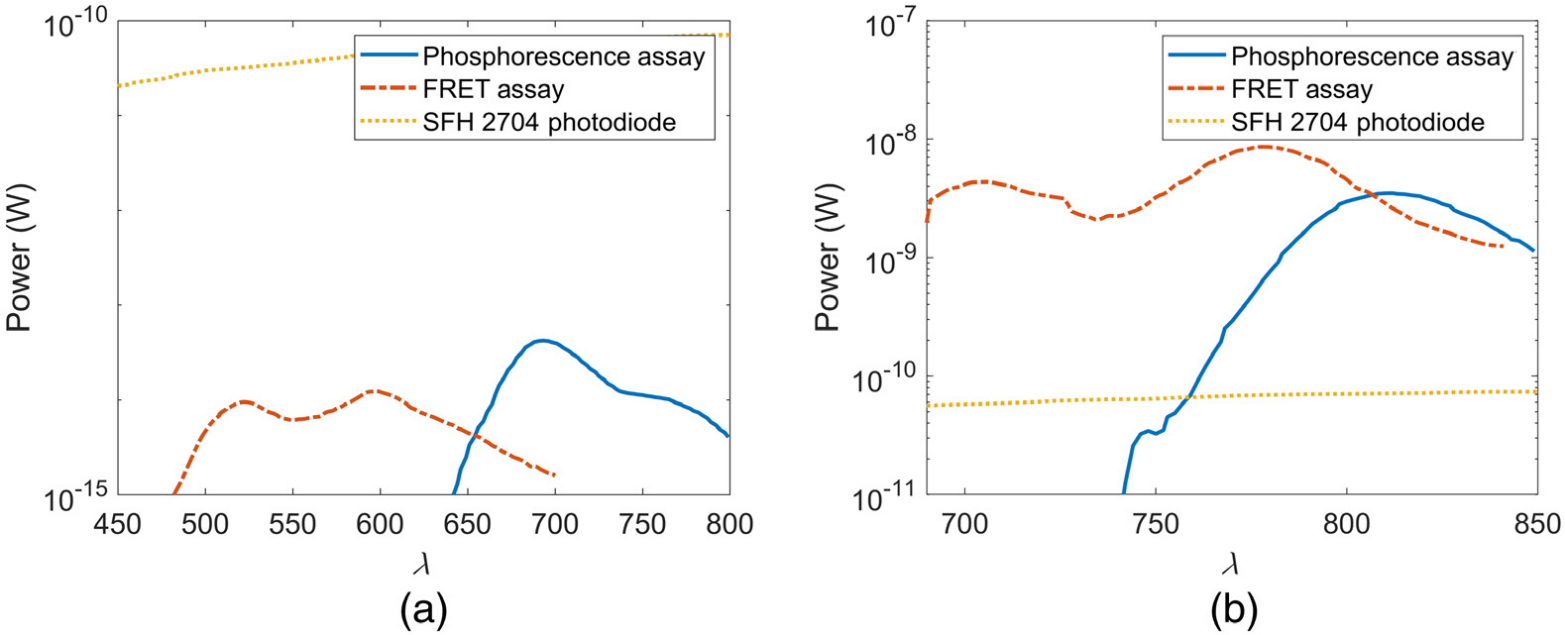
Comparison of assay power at the detector for a 32-mW excitation LED to SFH 2704 photodiode minimum detectable power. (a) Visible spectrum biosensor and (b) far-red biosensor.

As light penetration through tissue generally increases as a function of wavelength within the visible spectrum, it is anticipated that the far-red version of the barcode will have greater output power. Additionally, the higher molar extinction coefficients of far-red dyes used in this work compared with their visible spectrum counterparts allow for an increase in light absorption and thus light emission. Figure 11 illustrates not only the change in observed power with each assay but also the detectability on a low-cost photodiode commonly used in biosensing (SFH 2704). In each case the simulated source power is 32 mW, which was chosen because it achieves the maximum $2\;\mathrm{kW}/m^{2}$ permissible exposure for 450 and 680 nm. Figure 11(a) shows that the visible spectrum assays emit light such that only a maximum of $1.24\times 10^{-14}\;W$ and $4.23\times 10^{-14}\;W$ of light reaches the photodetector aperture for the FRET and phosphorescence assays, respectively. This value is too low to be detectable by the majority of low-cost photodiodes. Additionally, this assay combination requires a wide window of detectability, which would increase the photodiode cost when such a significant minimum detectable power is required. The far-red assay demonstrates an increased power output up to five orders of magnitude greater, as shown in Fig. 11(b). The greatest magnitude expected is $8.58\times 10^{-9}\;W$. It is also found that the fluorescence output of the far-red FRET assay is one order of magnitude greater than the simulated fluorescence output of the endogenous melanin. However, these conditions assume the near-ideal optical conditions of superficial implant depth, low volume fraction melanosomes, and colocated source and detector.

**Fig. 12:**
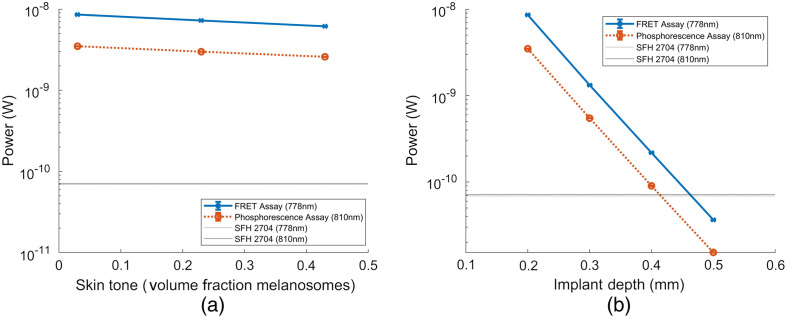
Parametric sweep of assay power output at a wavelength of maximum intensity. (a) Varying implant depth from 0.30 to 0.50 mm and (b) varying skin tone from 0.03 volume fraction melanosome (∼Fitzpatrick skin tone I) to 0.43 volume fraction melanosome (∼Fitzpatrick skin tone VI).

Figure 12 shows the decrease in photoluminescent intensity of each assay’s maximum emission wavelength, 778 and 810 nm for the FRET and phosphorescence lifetime assay, respectively, as a function of implant depth and increase in melanin concentration. Figure 12(a) shows that at the peak emission wavelength, both assays remain detectable from an approximation of Fitzpatrick skin tone I through Fitzpatrick skin tone VI. The percent decrease in signal intensity observed in both assays is 28.5% and 25.9% for the FRET and phosphorescence assay, respectively. Figure 12(b) shows that, at the peak emission wavelength, both assays remain detectable until implantation depth is 0.50 mm. As implant depth increases by 0.1 from 0.20 to 0.50 mm, signal intensity decreases anywhere from 83% to 85% for each iteration. The COV when repeating these simulations in triplicate increases from 0.2% to 0.7%, indicating that 1e7 photons is still sufficient for making these measurements.

Overall, the simulations indicate that both the FRET and phosphorescence assays are detectable by this photodiode, but the signal-to-noise ratio is low, especially in cases of deeper implantation and dark skin tone. Thus, future work will involve maximizing the fluorescence output of this biosensor. Additionally, this work features a colocated source and detector. This was done to properly validate these computational models, as an offset between source and detector for the barcode, which does not have rotational symmetry at 90 deg and 180 deg, would lead to spectral changes as smaller-wavelength components of emission spectra will not propagate as far as larger-wavelength components of emission spectra. In practice, this colocated configuration is unlikely to be achievable, and the addition of spacing between the source and detector would reduce the power output of this assay. To this end, it is important to determine an ideal source/detector configuration in follow-up studies.

## 4 Conclusion

We created a Monte Carlo framework using MCmatlab that predicts the fluorescent output of a fully insertable and multimodal glucose biosensor. The results of this framework were compared against visible wavelength results reported in previous literature and then expanded to estimate the fluorescence output of a biosensor with different assay components and in the red to NIR range. We were able to represent tissue autofluorescence and to quantify the expected fluorescent power output of the red-NIR light actuated barcode to be five orders of magnitude greater than a visible spectrum excited counterpart biosensor. This framework was applied to conceptualize a novel “barcode” glucose biosensor that uses repeating domains of two different sensing modalities, and it was found that such a biosensor should yield an output strong enough to be detectable by an off-the-shelf photodiode. This framework can be used to develop glucose biosensors that use either FRET and/or phosphorescence lifetime decay assays.

## Code, Data, and Materials Availability

The code used to generate the results is available in a GitHub repository: https://github.com/TEES-CRHTS/montecarlobarcode.
