# Supplementary material for: Monte Carlo method for assessment of a multimodal insertable biosensor
Source: J Biomed Opt. 2022 May 3;27(8):083017. doi: 10.1117/1.JBO.27.8.083017 (PMC9064117; doi:10.1117/1.JBO.27.8.083017)
Supplement: Supplementary file 1 [file JBO_027_083017_SD001.pdf]

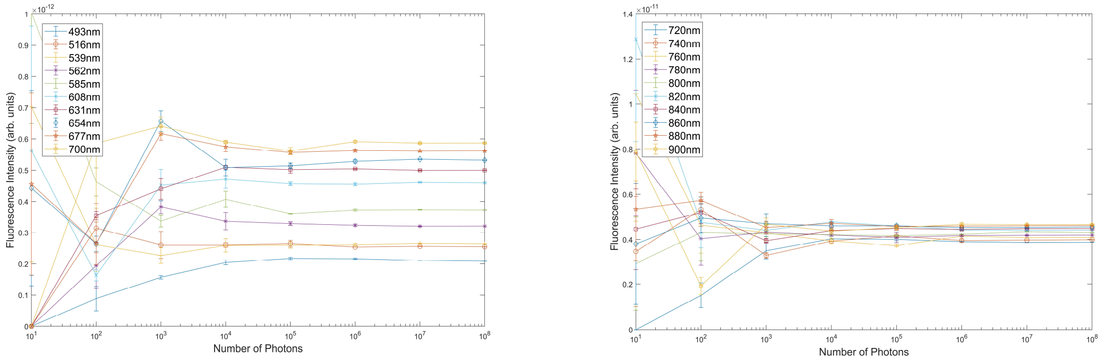

(a) (b)

Fig. S1 (a) Parametric Convergence for 450nm-excited Monte Carlo Simulations (b) Parametric Convergence for 680nm-excited Monte Carlo Simulations

Supplementary Note S1. Explanation of Parametric Analysis

Supplementary Fig. S1 shows parametric convergence for two biosensor iterations: one with an excitation of 450nm and one with an excitation of 680nm. As described in section 2.6, excitation and emission simulations with a target fluorophore of absorption coefficient and power yield of 0.001cm^{-1} and 0.001 respectively were carried out with increasingly many photons in triplicate. It was found that the fluorescence intensity reaching the detector when 10^7 photons were used was within 5% of the fluorescence intensity reaching the detector when 10^8 photons were used, indicating that simulation output has converged at 10^7 photons in both cases.

Supplementary Note S2. Number of Simulations Required for a Single Barcode Biosensor Iteration

Phosphorescence Lifetime Assay:

1. 1 excitation simulation (x3 for triplicate. 3 total)
2. 80 emission simulations (x3 for triplicate, 240 total)

FRET Assay

1. 1 excitation simulation (x3 for triplicate, 3 total)
2. 80 donor dye emission simulations (x3 for triplicate, 240 total)
3. 80 acceptor dye emission simulations (x3 for triplicate, 240 total)
